# Influence of intraoperative findings on immediate flow through radial-cephalic arteriovenous wrist fistulas for hemodialysis access

**DOI:** 10.1590/1677-5449.001518

**Published:** 2018

**Authors:** Afonso César Polimanti, Rafael Vilhena de Carvalho Fürst, Sidnei José Galego, Alexandre Sacchetti Bezerra, Fernando Adami, João Antônio Corrêa

**Affiliations:** 1 Faculdade de Medicina do ABC, Disciplina de Angiologia e Cirurgia Vascular, Santo André, SP, Brasil.; 2 Faculdade de Medicina do ABC, Disciplina de Fundamentos de Cirurgia, Santo André, SP, Brasil.; 3 Faculdade de Medicina do ABC, Laboratório de Epidemiologia e Análise de Dados, Santo André, SP, Brasil.

**Keywords:** renal dialysis, risk factors, arteriovenous shunt, surgical/adverse effects/methods, predictive value of tests, thrombosis/diagnosis/etiology/physiopathology, diálise renal, fatores de risco, derivação arteriovenosa, cirúrgica/efeitos adversos/métodos, valor preditivo dos testes, trombose/diagnóstico/etiologia/fisiopatologia

## Abstract

**Background:**

Adequate flow through a newly created arteriovenous fistula depends on multiple characteristics of the vessels and patient comorbidities. Several studies have related preoperative findings to failure, but few have analyzed the influence of intraoperative findings.

**Objectives:**

To evaluate the predictive value of intraoperative findings on the immediate outcome of radial-cephalic arteriovenous wrist fistulas (RCAVF) by collecting data that are easily measured intraoperatively.

**Methods:**

We designed a cross-sectional study, in which a single surgeon performed 101 RCAVF in 100 patients at a single center. We analyzed the immediate postoperative flow, assessed by thrill intensity immediately after fistula creation, against patient demographics and intraoperative data. The following variables were analyzed: age, sex, comorbidities, length of vein visible at preoperative examination, macroscopic arterial calcification, maximum vein diameter, and length of stenosis-free vein, measured by cannulation with a urethral catheter during the procedure. The chi-square test was used both to eliminate possible bias introduced by side of venous access (left or right), and to determine predictive values of immediate thrill.

**Results:**

Side of access was not associated with any significant differences in variables. Absence of macroscopic arterial calcification, successful venous catheterization using a 6 French catheter or larger, and ability to advance it more than 10 centimeters along the lumen of the proximal vein were correlated with adequate immediate postoperative thrill (p = 0.004, p < 0.001, and p = 0.005, respectively).

**Conclusions:**

In this series of 101 RCAVF, both the diameter of the catheter and its progress through the proximal vein and also absence of arterial calcification had positive predictive value for achieving adequate immediate thrill after vascular access construction.

## Resumo

**Contexto:**

O fluxo adequado de uma fístula arteriovenosa recém-confeccionada depende de múltiplas características dos vasos, bem como de comorbidades do paciente. Diversos estudos associam achados pré-operatórios e suas relações à falha do acesso, mas poucos analisam a influência dos achados encontrados no intraoperatório.

**Objetivos:**

Avaliar o valor preditivo desses achados no fluxo imediato de fístulas arteriovenosas radiocefálicas de punho (FAVRCPs) para hemodiálise através de coleta de dados facilmente mensuráveis do intraoperatório.

**Métodos:**

Um único cirurgião realizou 101 FAVRCPs em 100 pacientes, em um único centro. Analisou-se o fluxo do pós-operatório imediato através da intensidade do frêmito imediatamente após a confecção do acesso, e os resultados foram comparados com dados demográficos e achados intraoperatórios. As variáveis analisadas foram sexo, idade, comorbidades, extensão da veia visível ao exame físico, presença de calcificação arterial macroscópica, diâmetro venoso máximo e extensão de veia livre de estenose, avaliadas através da cateterização venosa com sonda uretral durante o procedimento. O teste de qui-quadrado foi utilizado tanto para excluir um possível viés de confusão pelo lado do acesso como para avaliar o valor preditivo das variáveis na intensidade do frêmito.

**Resultados:**

Não foi identificada diferença significativa nas variáveis segundo o lado do acesso. A ausência de calcificação arterial, a possibilidade de cateterização venosa com sonda de 6 French ou maior, e a sua progressão por mais de 10 cm através do lúmen da veia proximal tiveram correlação com frêmito imediato adequado ao fim do procedimento (p = 0,004, p < 0,001 e p = 0,005, respectivamente).

**Conclusões:**

Nesta série de 101 FAVRCPs, o diâmetro e a progressão de cateter 6 French ou maior através da veia proximal e a ausência de calcificação arterial apresentaram valor preditivo positivo na obtenção de frêmito imediato adequado de FAVRCP para hemodiálise.

## INTRODUCTION

 Creation of a chronic vascular access for hemodialysis (HD) became reality in 1960 and was the result of the combined efforts of a surgeon and a nephrologist, who designed a U-shaped device that allowed both arterial and venous access, making hemodialysis feasible for end-stage kidney disease (ESKD) patients. 1 While the Quinton-Scribner shunt was a breakthrough for ESKD treatment, it was associated with a high incidence of complications, mainly involving thrombosis and infections. 2

 In 1966, Michael Brescia and James Cimino first described creation of a radial-cephalic arteriovenous wrist fistula (RCAVF) to provide access for HD, offering greater patency and lower risk of complications. 3 Today, fifty years after that pioneering report, the RCAVF is still the access of choice for ESKD when anatomy is suitable. 4 Over the years, technical aspects of arteriovenous fistulas (AVF) have been reported that make long-term HD feasible, depending on inherent patient characteristics. 5 ^-^ 8

 Although RCAVF is the first option for ESKD, it has a high rate of failure to mature, which can be as high as 40%. 9 Adequate flow rates have a direct influence on the effectiveness of HD and, therefore, on morbidity and mortality of ESKD patients. 4 ^,^ 10 Several studies have analyzed the influence of preoperative examination and vein mapping on the patency of AVF. 11 ^-^ 14

 However, irrespective of preoperative investigations, surgeons are sometimes faced with intraoperative findings that differ from preoperative imaging exams, creating a need for studies of the impact that variables that can be identified during surgery have on the outcome of the procedure, enabling better decision making during surgery. 

 Auscultation of bruit with a stethoscope and the tactile sensation of thrill on palpation of AVF immediately after creation are related to spiral laminar flow through the vessel, which is associated with a higher probability of correctly maturing AVF. 15 ^-^ 18 Thrill intensity is the only one of these parameters that can be acted upon when observed during the surgical procedure. 

 We have previously published a pilot analysis of 39 AVF, including distal and upper arm AVF, venous transpositions, and an arteriovenous graft, evaluating intraoperative findings that are easily obtained during the procedure and their impact on immediate venous access thrill. Although it was a small series, we found statistically significant correlations for satisfactory immediate thrill and the feasibility of cannulating the vein in a segment exceeding 10 centimeters in length. 19 Since that first study included multiple different types of surgical accesses, confidence in the results may not be reliable, due to possible bias from confounding variables, such as which side the procedure is conducted on, and the different flow patterns at different access sites. We have therefore chosen to focus on a single type of AVF, the RCAVF. 

 The aim of the present study is to evaluate the predictive value of intraoperative findings easily acquirable during the procedure on the thrill assessed immediately after creation of RCAVF. 

## METHOD

 This is a cross-sectional study. Data were collected from 100 patients with ESKD, all scheduled to undergo RCAVF creation, performed by the same surgeon (ACP), at the ABC Integrated Center of Nephrology (CINABC) from 2012 to 2015. 

 The inclusion criteria were all patients with ESKD with technical feasibility for RCAVF creation. The exclusion criteria were a positive Allen’s test and absence of a suitable cephalic vein at wrist level during the preoperative physical examination. 

### Preoperative assessment

 All patients underwent Allen’s test and venous mapping by physical examination, placing a tourniquet on the proximal forearm, immediately distal to the cubital fossa, while the patient clenched and released the ipsilateral hand several times. This maneuver enabled observation of the maximum extension of cephalic vein visible above the wrist. Ultrasound venous mapping was not performed as routine, because of lack of access to our patients to perform duplex scanning. 

### Surgical procedure

 All procedures were undertaken under local anesthesia, with 2% Lidocaine solution via a longitudinal incision. The cephalic vein was cannulated with a urethral catheter, made of soft polyvinyl chloride (PVC), with a size ranging from 4 to 10 French (Fr), which was used to measure the vein diameter and dilate the vessel with saline solution. This is a common practice at our institution, because there is less intraluminal trauma, when compared to coronary probes. We then measured the length of stenosis-free vein by introducing the catheter into the vein lumen, and classified the patient into one of two groups: Group I, if the catheter advanced more than 10 centimeters (cm), or Group II, if the catheter could not be advanced by 10 cm. 

 The radial artery was exposed with minimal dissection; arterial flow was interrupted by traction of silicone ribbons, followed by longitudinal arteriotomy and venotomy. At this point of the procedure, macroscopic arterial calcification is identified if present. An arteriovenous anastomoses was constructed with continuous 6-0 or 7-0 polypropylene suture, by a modified lateral-lateral technique, as described by Galego et al. 20 Immediately after completion of the anastomosis, the immediate flow in the venous access is evaluated by grading thrill at the anastomosis region, defining it as absent, weak, medium, or strong thrill. Weak and negative thrills were grouped as inadequate thrill, and medium and strong thrills were classified as adequate thrill. 

### Variables evaluated

 The following variables were analyzed: side of AVF creation, comorbidities, vein length at physical examination, diameter of urethral catheter used during procedure, progress of catheter inside vein lumen, presence of arterial calcification, and thrill intensity identified immediately after completion of the procedure. 

### Ethics

 Informed consent was obtained from all patients prior to the procedure, and the Medical Ethics Committee of Heliopolis Hospital approved the study, registered as CEP 809, under protocol number 443457. 

### Statistical analysis

 In order to avoid confounding bias caused by anatomic differences between right and left arms, all variables were compared by side of AVF creation, using the chi-square test. They were later compared by thrill intensity after the procedure, using univariate analysis with the chi-square test. Results with p-values below 0.05 were considered statistically significant. Analyses were performed using STATA software version 1. 

## RESULTS

 From 2012 to 2015, 100 patients were enrolled on the study. A total of 101 RCAVF were performed at CINABC. Demographic data for the subjects are given in Table 1 . The majority of the patients were male (73.27%), and the most frequent etiologies of kidney failure were hypertension and diabetes. Mean age was 60.19 years, and 42.57% of patients were over the age of 65 years. Diabetes was defined as adult onset, controlled with diet or insulin, or juvenile onset as described by SVS reporting standards. Similarly, hypertension was defined as diastolic blood pressure greater than 90 mmHg, or blood pressure controlled with one or more anti-hypertensive medications. One patient underwent procedures on both sides, due to early thrombosis of the venous access. 

**Table 1:** Demographic and clinical data on the study population.

**Characteristics**	**Number (%)**
Total RCAVF in analysis	101
Side of AVF	
Left	71 (70.3%)
Right	30 (29.7%)
Age	
Mean	60,19
Over 65	43
Gender	
Male	74 (73.27%)
Female	27 (26.73%)
Comorbidities	
Hypertension	78.22%
Diabetes mellitus	42.57%
Other ^*^	23.76%

* Other: Glomerulonephritis, Congestive heart failure, Viral Hepatitis, History of Human Immunodeficiency Virus, Systemic lupus erythematosum, post renal obstruction, non steroidal analgesics toxicity.

 Most of the procedures were performed on the left arm (70.3%). The chi-square test detected no statistically significant differences in any of the anatomical variables associated with the side on which the procedure was performed, as shown in Table 2 . This enabled us to analyze all procedures together without considering the side. 

**Table 2:** Anatomical comparison by side of procedure.

**Characteristics**	**Right**	**Left**	***p*-value**
Vein extension at PE			
1	7	10	0.626
2	6	20	
3	10	22	
4	7	19	
Vein Caliber			
4Fr	1	3	0.074
6Fr	16	53	
8Fr	13	15	
Catheter Progression			
Less than 10 cm	4	15	0.360
More than 10 cm	26	56	
Calcification			
Present	20	46	0.856
Absent	10	25	
Thrill Intensity			
Adequate	26	58	0.541
Inadequate	4	13	

Fr = French; PE = physical examination.

 An adequate immediate thrill was achieved in 84% of the procedures. The remaining 16% underwent upper-arm AVF creation during the same operation. Table 3 summarizes the analysis of intraoperative findings with relation to immediate postoperative thrill. Presence of arterial calcification visible macroscopically during the procedure is associated with inadequate flow through the venous access immediately after anastomosis creation (p = 0.04). 

**Table 3:** Immediate postoperative flow, by preoperative and intraoperative findings.

**Characteristics**	**Inadequate thrill**	**Adequate thrill**	***p*-value**
Age			
Under 65	9	49	0.682
Over 65	8	35	
Sex			
Male	12	62	0.784
Female	5	22	
Diabetes Mellitus	9	34	0.343
Hypertension	12	67	0.403
Vein extension at PE			
1	4	13	0.214
2	6	20	
3	6	26	
4	1	25	
Vein Caliber			
4Fr	3	1	0.005 ^*^
6Fr	11	58	
8Fr	3	25	
Catheter Progression			
< 10 cm	11	8	< 0.001 ^*^
> 10 cm	6	76	
Calcification			
Present	11	24	0.004 ^*^
Absent	6	60	

_*_ Statistical significance on chi-square test.

 It was also observed that successful venous catheterization using a 6Fr catheter or larger and ability to advance it along more than 10 centimeters of stenosis- free lumen were related to higher intensity of thrill at the end of the procedure (p = 0.005 and p < 0.01, respectively). 

## DISCUSSION

 The main goal of AVF creation is to obtain a venous access that allows repeated cannulation, providing sufficient flow to perform adequate dialysis sessions on a long-term basis. Although an RCAVF is considered the gold standard for hemodialysis access, because of its lower blood flow compared to an upper arm AVF, the literature reports high rates of failure to mature, ranging from 9% to 40%. 21 ^,^ 22 In our series, inadequate flow was found in 16.83%. 

 According to Kidney Disease Outcome Quality (KDOQI) guidelines, the main etiologies of ESKD are hypertension and diabetes. 4 This was confirmed in our findings, as these conditions were present in 79% and 42.57%, respectively. 

 In the literature, female gender, increasing age, and diabetes mellitus are related to poor results. 6 ^,^ 8 ^,^ 23 ^,^ 24 Jemcov et al. found that females had smaller arteries and that radial-cephalic AVF took longer to mature in women, but they had higher venous access flow compared to men. 13 Diabetes has been associated with poor AVF results, and lower blood flow, probably due to its influence on accelerating atherosclerosis progression on distal arteries. 8 ^,^ 22 ^,^ 25 In our series, these factors had no significant influence on AVF outcome. 

 Although increasing age is related to poor-quality veins, more advanced stages of atherosclerosis, and higher incidence of comorbidities, there is no consensus on whether age is related to AVF failure. The AVF recommendations in KDOQI guidelines do not distinguish between younger and older patients. 4 ^,^ 26 Some studies report higher failure rates in patients over the age of 80 years, 27 whereas other studies have not found proof of association between age-related risk factors and fistula failure. 26 In our series, 42.58% of procedures were performed in patients over the age of 65 years, and no effect on immediate outcome was observed. 

 Measurements of intraoperative flow have prognostic value with relation to AVF patency. A variety of methods for taking these measurements have been described, including Doppler ultrasound, handheld ultrasound flow meters, 28 electromagnetic flow meters, 20 and magnetic resonance imaging. 29

 It is well known that venous access flow is higher through proximal AVF when compared to RCAVF. 30 In our study, we chose to only evaluate RCAVF, to avoid data collection confounding bias. 

 Computer simulated flow studies illustrate that an adequately functioning AVF has a spiral flow pattern. 31 During physical examination, this flow pattern is related to a palpable thrill, which in turn is related to adequate flow through the venous access. 16 ^,^ 28 It can be easily evaluated during the procedure, with no need for any extra equipment for analysis. 16 In order to increase the reliability of measurement comparisons, in the present study all procedures were performed by a single surgeon. 

 Although thrill intensity immediately after AVF creation is assessed subjectively and some studies describe a lack of correlation with fistula maturation, 5 ^,^ 18 several studies rely on this parameter to evaluate access outcomes. 19 ^,^ 22 ^,^ 24 ^,^ 32 ^,^ 33

 It is known that atherosclerosis and arterial calcification alter arterial elasticity. 34 In their studies, Young 35 and Kim 36 noted a higher rate of early failure in forearm AVF created in patients with intimal hyperplasia and calcification. Kheda stated that these findings led to reduced arterial elasticity, which is also associated with maturation failure. It is known that diabetes is related to medial calcific sclerosis, Monckeberg’s arteriosclerosis, which may also play a role in reducing vessel elasticity. 37 In the present study, macroscopic arterial calcification had a statistical correlation with inadequate flow (p = 0.004), but diabetes mellitus was not associated with early failure (p = 0.343). 

 Several studies discuss the minimum venous diameter for AVF creation. Some series describe physical examination as an accurate method of preoperative assessment, but the methods used are distinct for each study and a high degree of subjectivity in vein diameter measurement and venous segment assessment make them difficult to reproduce. 38 In our study, physical examination assessments had no statistically significant relationship with the outcome of the procedure. 

 The results of Doppler ultrasound studies of minimum vein diameter range from 1.8mm to 4.0mm. 7 ^,^ 13 ^,^ 14 ^,^ 22 ^,^ 39 Fila suggested dilating veins during the procedure, measuring the vein using probes gently inserted before anastomosis, finding that results were better with dilated veins of diameter greater than 3.5mm. 40 The catheter method used in our study is similar to intraoperative measures described by Fila. 40 Urethral catheters have the advantage of being widely available compared to vessel probes, and sizes are easily comparable with ultrasound mapping, since urethral catheters of 4Fr, 6Fr, 8Fr and 10Fr are equivalent to probes of 1.33mm, 2mm, 2.66mm and 3.33mm, respectively. Cannulation with soft PVC urethral catheters and dilatation by saline infusion are also less likely to cause inadvertent vein rupture when compared to forcing the diameter to expand exclusively through vessel cannulation with rigid structures during the procedure. 

 We observed better results with catheters of 6Fr or larger (or 2mm, converting French units to millimeters). This data is similar to direct venous measurement by probes, 40 and ultrasound mapping studies, 12 ^,^ 14 which report better results from AVF created with veins with a minimum preoperative diameter of 2mm. 

 Fila also reported venous cannulation of 10 to 15 cm proximal to the anastomosis site, yet this length was not evaluated in detail as a variable possibly associated with access outcome. 40 KDOQI suggests an adequate AVF should have at least 6 to 10 cm of vein available to allow rotation of puncture sites. 4 We found better outcomes for RCAVF in which it was possible to perform venous cannulation along at least a 10 cm segment of vein (p < 0.001). 

 This study has limitations. Although all the procedures and flow analysis were performed by a single surgeon, on a single type of AVF, measurement of flow by thrill palpation is subjective. Both thrill intensity and venous diameter can be underestimated due to vessel spasm secondary to manipulation. A longitudinal study is needed to compare the intraoperative findings to actual access functionality and to analyze the correlation between immediate thrill palpation and AVF outcome. Further studies are needed to evaluate whether these variables identified during surgery actually have an influence on maturation time and dialysis access performance. 

## CONCLUSION

 The present study shows that absence of calcification, feasibility of venous cannulation with a urethral catheter of a diameter greater than 2 mm (6Fr), and venous cannulation of more than 10 cm are associated with better AVF outcomes. Careful intraoperative venous measurements may provide more information to help vascular surgeons to make the decision on whether it is better to explore more proximal access possibilities during surgery or to create an AVF at the site originally intended. 

## References

[1] Quinton W, Dillard D, Scribner BH (1960). Cannulation of blood vessels for prolonged hemodialysis. Trans Am Soc Artif Intern Organs.

[2] Foran RF, Golding AL, Treiman RL, De Palma JR (1970). Quinton-scribner cannulas for hemodialysis. Review of four years’ experience. Calif Med.

[3] Brescia MJ, Cimino JE, Appel K, Hurwich BJ (1966). Chronic hemodialysis using venipuncture and a surgically created arteriovenous fistula. N Engl J Med.

[4] National Kidney Foundation (2006). KDOQI Clinical Practice Guidelines and Clinical Practice Recommendations for 2006 Updates: Hemodialysis Adequacy, Peritoneal Dialysis Adequacy and Vascular Access. Am J Kidney Dis.

[5] Wong V, Ward R, Taylor J, Selvakumar S, How TV, Bakran A (1996). Factors associated with early failure of arteriovenous fistulae for haemodialysis access. Eur J Vasc Endovasc Surg.

[6] Hodges TC, Fillinger MF, Zwolak RM, Walsh DB, Bech F, Cronenwett JL (1997). Longitudinal comparison of dialysis access methods: risk factors for failure. J Vasc Surg.

[7] Lauvao LS, Ihnat DM, Goshima KR, Chavez L, Gruessner AC, Mills JL (2009). Vein diameter is the major predictor of fistula maturation. J Vasc Surg.

[8] Cruz RN, Retzlaff G, Gomes RZ, Reche PM (2015). Influência do diabetes mellitus sobre a perviedade da fístula arteriovenosa para hemodiálise. J Vasc Bras.

[9] Tordoir JHM, Rooyens P, Dammers R, van der Sande FM, de Haan M, Yo TI (2003). Prospective evaluation of failure modes in autogenous radiocephalic wrist access for haemodialysis. Nephrol Dial Transplant.

[10] Neves MA, Petnys A, Melo RC, Rabboni E (2013). Acesso vascular para hemodialise: o que ha de novo?. J Vasc Bras.

[11] Leon C, Asif A (2008). Physical examination of arteriovenous fistulae by a renal fellow: does it compare favorably to an experienced interventionalist?. Semin Dial.

[12] Glass C, Johansson M, DiGragio W, Illig KA (2009). A meta-analysis of preoperative duplex ultrasound vessel diameters for successful radiocephalic fistula placement. Journal for Vascular Ultrasound..

[13] Jemcov TK (2013). Morphologic and functional vessels characteristics assessed by ultrasonography for prediction of radiocephalic fistula maturation. J Vasc Access.

[14] Bashar K, Clarke-Moloney M, Burke PE, Kavanagh EG, Walsh SR (2015). The role of venous diameter in predicting arteriovenous fistula maturation: when not to expect an AVF to mature according to pre-operative vein diameter measurements? A best evidence topic. Int J Surg.

[15] Saucy F, Haesler E, Haller C, Déglise S, Teta D, Corpataux JM (2010). Is intra-operative blood flow predictive for early failure of radiocephalic arteriovenous fistula?. Nephrol Dial Transplant.

[16] Marie Y, Guy A, Tullett K, Krishnan H, Jones RG, Inston NG (2014). Patterns of blood flow as a predictor of maturation of arteriovenous fistula for haemodialysis. J Vasc Access.

[17] Jamil M, Usman R (2015). Predictive parameters for successful functional maturation of native arteriovenous fistula. J Ayub Med Coll Abbottabad.

[18] Srivastava A, Mittal V, Lal H (2015). Spiral laminar flow, the earliest predictor for maturation of arteriovenous fistula for hemodialysis access. Indian J Urol.

[19] Galego SJ, Corrêa JA, Yamazaki YR (2006). Estudo retrospectivo da influência dos parâmettros intra-operatórios no valor preditivo do fluxo imediato de fístulas arteriovenosas realizadas para acesso de hemodiálise. Rev Méd IAMSPE..

[20] Galego SJ, Goldenberg S, Ortiz JP, Deoliveira Gomes P, Ramacciotti E (2000). Comparative blood flow study of arteriovenous fistulae in canine femoral arteries: modified latero-lateral and end-lateral techniques. Artif Organs.

[21] Centofanti G, Fujii EY, Cavalcante RN (2011). An experience of vascular access for hemodialysis in Brazil. Int Arch Med.

[22] Sahasrabudhe P, Dighe T, Panse N, Deshpande S, Jadhav A, Londhe S (2014). Prospective long-term study of patency and outcomes of 505 arteriovenous fistulas in patients with chronic renal failure: Authors experience and review of literature. Indian J Plast Surg.

[23] Huber TS, Ozaki CK, Flynn TC (2002). Prospective validation of an algorithm to maximize native arteriovenous fistulae for chronic hemodialysis access. J Vasc Surg.

[24] Sahasrabudhe P, Dighe T, Panse N, Patil S (2013). Retrospective analysis of 271 arteriovenous fistulas as vascular access for hemodialysis. Indian J Nephrol.

[25] Prischl FC, Kirchgatterer A, Brandstätter E (1995). Parameters of prognostic relevance to the patency of vascular access in hemodialysis patients. J Am Soc Nephrol.

[26] Olsha O, Hijazi J, Goldin I, Shemesh D (2015). Vascular access in hemodialysis patients older than 80 years. J Vasc Surg.

[27] Patel ST, Hughes J, Mills JL (2003). Failure of arteriovenous fistula maturation: an unintended consequence of exceeding dialysis outcome quality Initiative guidelines for hemodialysis access. J Vasc Surg.

[28] Won T, Jang JW, Lee S, Han JJ, Park YS, Ahn JH (2000). Effects of intraoperative blood flow on the early patency of radiocephalic fistulas. Ann Vasc Surg.

[29] Gonzalez AJ, Casey KM, Drinkwine BJ, Weiss JS (2016). Series of noncontrast time-of-flight magnetic resonance angiographies to identify problems with arteriovenous fistula maturation. Ann Vasc Surg.

[30] Polkinghorne KR, Atkins RC, Kerr PG (2004). Determinants of native arteriovenous fistula blood flow. Nephrology (Carlton).

[31] Bessa KL, Ortiz JP (2009). Flow visualization in arteriovenous fistula and aneurysm using computational fluid dynamics. J Vis (Tokyo).

[32] Trerotola SO, Ponce P, Stavropoulos SW (2003). Physical Examination versus Normalized Pressure Ratio for Predicting Outcomes of Hemodialysis Access Interventions. J Vasc Interv Radiol.

[33] Corrêa JA, Pires AC, Kafejian O (2005). Superficial saphenofemoral arteriovenous fistula as access to hemodialysis - Description of operative technique and initial clinical experience. J Vasc Bras.

[34] Karwowski W, Naumnik B, Szczepanski M, Mysliwiec M (2012). The mechanism of vascular calcification - a systematic review. Med Sci Monit.

[35] Kim YO, Choi YJ, Kim JI (2006). The impact of intima-media thickness of radial artery on early failure of radiocephalic arteriovenous fistula in hemodialysis patients. J Korean Med Sci.

[36] Kim YO, Song HC, Yoon SA (2003). Preexisting intimal hyperplasia of radial artery is associated with early failure of radiocephalic arteriovenous fistula in hemodialysis patients. Am J Kidney Dis.

[37] Kheda MF, Brenner LE, Patel MJ (2010). Influence of arterial elasticity and vessel dilatation on arteriovenous fistula maturation: a prospective cohort study. Nephrol Dial Transplant.

[38] Nursal TZ, Oguzkurt L, Tercan F (2006). Is routine preoperative ultrasonographic mapping for arteriovenous fistula creation necessary in patients with favorable physical examination findings? Results of a randomized controlled trial. World J Surg.

[39] Robbin ML, Chamberlain NE, Lockhart ME (2002). Hemodialysis arteriovenous fistula maturity: US evaluation. Radiology.

[40] Fila B, Lovcic V, Sonicki Z, Magas S, Sudar-Magas Z, Malovrh M (2014). Vein diameter after intraoperative dilatation with vessel probes as a predictor of success of hemodialysis arteriovenous fistulas. Med Sci Monit.

